# Role of lipins in cardiovascular diseases

**DOI:** 10.1186/s12944-023-01961-6

**Published:** 2023-11-14

**Authors:** Zerui Ding, Hongyu Song, Fang Wang

**Affiliations:** 1 The Endocrinology Department of the Third Xiangya Hospital, Central South University, Changsha, 410013 China; 2 Xiangya School of Medicine, Central South University, Changsha, 410013 China

**Keywords:** Lipin, Cardiovascular diseases, Inflammation, Lipid metabolism, Triacylglycerol

## Abstract

Lipin family members in mammals include lipins 1, 2, and 3. Lipin family proteins play a crucial role in lipid metabolism due to their bifunctionality as both transcriptional coregulators and phosphatidate phosphatase (PAP) enzymes. In this review, we discuss the structural features, expression patterns, and pathophysiologic functions of lipins, emphasizing their direct as well as indirect roles in cardiovascular diseases (CVDs). Elucidating the regulation of lipins facilitates a deeper understanding of the roles of lipins in the processes underlying CVDs. The activity of lipins is modulated at various levels, e.g., in the form of the transcription of genes, post-translational modifications, and subcellular protein localization. Because lipin characteristics are undergoing progressive clarification, further research is necessitated to then actuate the investigation of lipins as viable therapeutic targets in CVDs.

## Introduction

The marked prevalence of cardiovascular diseases (CVDs) has led to a heavy social burden on modern society, and this has long been the goal of medical research to optimize cardiovascular risk factors and targeted interventions to promote public health [1]. However, numerous risk factors for CVDs constitute a very complex network, including the interactions between related diseases and lifestyle and their influence on regulatory physiology. Thus, the exploration and research on the etiology, underlying mechanism(s) of action, and prevention of CVDs continue to advance [2].

Mammalian lipins, homologous to yeast Pah1p, are proteins that have Mg^2+^-dependent phosphatidate phosphatase (PAP) enzyme activity [3]. There are three members of the family, all of which show enzymatic activity as well as transcriptional coactivation activity [4]. Lipins regulate the balance between lipid synthesis and fatty acid catabolism, with the three lipin proteins exhibiting different expression patterns and functions within various tissues [5]. In addition to metabolic regulation, an increasing number of studies in recent years have revealed the functions of lipins in immunity and inflammation [6, 7]. Genetic defects or abnormal expression of lipins notably contribute to several conditions such as dyslipidemia, fatty liver, and altered inflammatory and immune response patterns [7]; and polymorphisms contribute to phenotypic heterogeneity, some of which are associated with increased susceptibility to CVDs [8]. In addition, some interventions with respect to CVDs exert modulatory effects on lipin-related pathways, tissue metabolism, and immune and inflammatory phenotypes, indicating a linkage between lipins and the complex network of risk factors for CVDs.

The roles of lipins in lipid metabolism and the immune system have been carefully reviewed previously [6, 7]. In this paper, we discuss our current knowledge pertaining to lipin functions and regulatory mechanisms as they relate to the pathophysiology of CVDs, and pharmacologic advances in the study of lipin-related drugs—focusing primarily on atherosclerosis and heart failure in studies from both rodents and humans.

## Multiple functions of lipins

### Structures of lipins

**Fig. 1:**
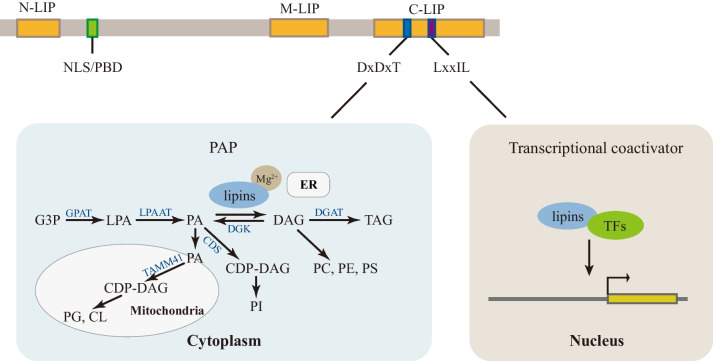
Schematic of the structure and function of lipins. NLS: nuclear localization signal; PBD: polybasic domain; N-LIP: amino-terminal lipin; C-LIP: carboxy-terminal lipin; M-LIP: middle lipin; PAP: phosphatidate phosphatase; G3P: glycerol 3-phosphate; GPAT: glycerol phosphate acyltransferase; LPA: lyso-phosphatidic acid; PA: phosphatidic acid; LPAAT: lyso-PA-acyltransferase; PLA: phospholipase A; DAG: diacylglycerol; DGAT: diacylglycerol acyltransferase; TAG: triacylglycerol; PC: phosphatidylcholine; PE: phosphatidylethanolamine; PS: phosphatidylserine; DGK: diacylglycerol-kinases; CDP-DAG: cytidine diphosphate diacylglycerol; CDS: CDP-DAG synthase; PI: phosphatidylinositol; PG: phosphatidylglycerol; CL: cardiolipin; and TFs: transcription factors

Lipin 1, lipin 2, and lipin 3 are identified as the three members of the lipin protein family. All family members contain several conserved regions, i.e., an amino-terminal domain (N-LIP), a carboxy-terminal lipin domain (C-LIP), and a middle lipin domain (M-LIP) [9, 10], and two key functional motifs within the C-LIP domain known as DxDxT and LxxIL. The DxDxT motif confers lipins with PAP enzyme activity, and the LxxIL motif is responsible for transcriptional coactivator activity [4, 11]. Recent studies have revealed that N-LIP co-folds with part of C-LIP with the help of M-LIP to form the functionally active PAP [10, 12]. In addition, lipins possess a conservative nuclear localization signal (NLS) or poly basic domain (PBD) that has lipid binding ability and is presumed to allow the translocation of lipins into the cell nucleus, where they interact with transcription factors and regulate gene expression (Fig. 1) [13].

### Expression patterns of lipins

**Fig. 2:**
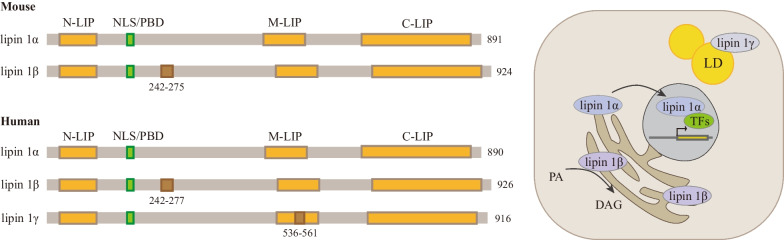
Structure and subcellular localization of lipin 1 isoforms in mice and humans. Lipin 1α mainly enters the nucleus as a transcriptional coactivator, while lipin 1β mainly acts as the cytoplasmic PAP enzyme on the endoplasmic reticulum and lipin 1γ is localized to lipid droplets to regulate their morphology during fatty acid stimulation

Three proteins are expressed differentially in various tissues, manifesting both unique and combined roles in certain contexts [14]. Lipin 1 is the most thoroughly studied subtype that attracts the greatest interest. In the wild-type mouse, lipin 1 is highly expressed in adipose tissue, skeletal muscle, testis, heart, and also in liver and other tissues [9]. Studies have confirmed the dual roles of lipin 1 in regulating metabolic balance by reflecting both PAP activity to catalyze phosphatidic acid (PA) conversion to diacylglycerol (DAG) (the penultimate step of triacylglycerol [TAG] synthesis in the Kennedy pathway) (Fig. 1), and coactivator activity to promote the expression of fatty acid oxidation (FAO) genes in liver and adipose tissue [4, 15]. Lipin 1, lipin 2, and lipin 3 seem to have redundancy in terms of PAP activity in some tissues, including in the liver and intestine [5]. In heart and skeletal muscle, lipin 1 is a determinant of PAP activity [14, 16]. Lipin 1 is a coactivator of multiple transcription factors, such as peroxisome proliferator-activated receptor α and γ (PPARα and PPARγ), PPARγ-coactivator 1α (PGC-1α), hepatocyte nuclear factor 4α (HNF4), and glucocorticoid receptor (GR) [4], of which the effects of lipin 1 on PPARγ are unique because lipin 1 has a transcriptional activation domain different from lipin 2 and lipin 3 [17]. Nuclear lipin 1 can also inhibit sterol-regulatory element binding proteins (SREBPs) and nuclear factor of activated T cells 4 (NFATc4), thus repressing downstream lipogenic gene and proinflammatory gene transcription, respectively [18, 19]. There are two mouse lipin 1 protein isoforms (lipin 1α and lipin 1β) and three human lipin 1 protein isoforms (lipin 1α, lipin 1β, and lipin 1γ), all of which have distinct subcellular localizations and functions. Lipin 1β has 33 and 36 more amino acids than lipin 1α in mice and humans, respectively, due to alternative splicing of the pre-mRNA. During adipogenesis, lipin 1α is localized to the nucleus and regulates the levels of adipogenic transcription factors PPARγ and C/EBP, while lipin 1β is predominantly cytoplasmic and mainly regulates lipogenesis in mature adipocytes [20]. Compared with lipin 1α, lipin 1γ has an insertion of 78 nucleotides close to its downstream flanking exon, and it has the lowest catalytic efficiency and fatty acyl specificity among the three isoforms. Lipin 1γ is localized to lipid droplets to regulate their morphology in response to fatty acid loading, and it is the mainly expressed isoform in the human brain (Fig. 2) [21, 22].

Majeed syndrome is an autoinflammatory disorder that manifests as chronic multifocal osteomyelitis, neutrophilic cutaneous inflammation, and dyserythropoietic anemia, which is caused by *LPIN2* mutation [23–25]. In studies of Majeed syndrome, lipin 2 was found to be expressed in numerous tissues [24]—most prominently in the liver, followed by kidney, lung, gut, and brain [25]. Lipin 2 is hypothesized to exhibit anti-inflammatory functions through PAP activity with respect to its expression in erythrocytes and lymphoid cells, the loss of which is postulated to contribute to the chief manifestations of Majeed syndrome that cannot be compensated for by lipin 1 and lipin 3 due to the much higher levels of lipin 2 within these tissues [25]. Lipin 1 and 2 work cooperatively to regulate hepatic PAP activity and TAG synthesis [26]. Similar to lipin 1, lipin 2 also exerts PAP and coactivator activities that are considered to be more important in the context of hepatic metabolism, given that its abundance in the liver is much higher than that of lipin 1 [25].

Lipin 3 is a relatively less-studied subtype that is principally expressed in trace amounts in the intestine, adipose tissue, and liver. Lipin 3 manifests PAP activity like other members of the lipin family [5], functions synergistically with lipin 1 in mouse adipogenesis and adiposity [14], and plays a significant role in chylomicron synthesis within intestinal enterocytes in cooperation with lipin 2 [27]. Lipin 3 can also interact with PPARα [4], but its specific action is poorly identified. Most recently, we found that haploinsufficiency of lipin3 in hepatocytes can upregulate lipin 1 expression, disrupt the nucleocytoplasmic localization of lipin 1, and affect mitochondrial function [28]. Subsequently, the balance between TAG anabolism and catabolism may be impaired through the regulation of lipin 1 phosphatidic acid phosphohydrolase enzyme activity and the lipin 1-PPARα-PGC1α pathway, further leading to HTG, NAFLD, and obesity.

It should be noted that lipins may have different affinities and catalytic activities toward different PAs (including palmitoyl, oleoylPA, and di-oleoylPA), and the catalytic efficiency of different types of lipins (lipin 1, lipin 2, and lipin 3 isoforms) on the same PA could also vary. Relevant studies are needed to further explore the possibilities.

## Functions of lipins in CVD-related tissues

The lipins manifest diverse expression patterns in various cell types, and researchers have depicted more precise profiles of lipins in disparate tissues [5] some of which are related to cardiovascular dysfunction and disease.

### Lipins in the heart: stress maladaptation and heart failure

Healthy cardiac muscles primarily use FAO for their energy supply, and normal lipid metabolism is vital for the health and function of the heart. In heart failure, cardiomyocytes reflect reduced mitochondrial oxidative capacity, attenuated FAO and glucose oxidation, and a compensatory increase in glycolysis. A mismatch between oxidative capacity and fatty acids can lead to lipotoxicity, leading to downstream signaling disturbances and an exacerbation in heart failure [29].

As a key factor regulating lipid metabolism, lipin 1 was found to be the primary PAP enzyme in the heart in several studies with rodents [14, 27, 30]. Fatty liver dystrophy (*fld*) mice that harbored a spontaneous *Lpin1* mutation exhibited life-long lipodystrophy and genetically programmed early postnatal fatty liver and hyperlipidemia [9, 31, 32]. *Fld* mice showed reduced systolic function, stroke volume, cardiac output, and pulmonary peak venous flows compared with age-matched control mice [33]. Nevertheless, the *fld* mice had 15%–20% reserve PAP activity in the heart and did not exhibit defective glycerolipid and phospholipid incorporation, indicating that other important pathways are involved in TAG synthesis in cardiomyocytes and that lipin 1 can be regarded as a cytosolic reservoir of PAP activity. Moreover, no significant differences were observed in glucose and oleate oxidation rates, or in cardiac function of *fld* mice hearts ex vivo. Thus, the cardiac dysfunction in *fld* mice found in vivo may be largely due to a systemic lack of lipin 1 [33]. Results of the effects of lipin 1 deficiency in neonatal rat ventricular myocytes are inconsistent, with some authors describing an acceleration of TAG synthesis [27], while others found no significant effect of either silencing or overexpressing lipin 1 [16].

To minimize interference by systemic lipin deficiency, investigators generated cardiac-specific lipin 1 knock-out mice. These mice showed an accumulation of cardiac PA, decreased cardiolipin levels, and reduced mitochondrial respiratory capacity. Although the mice did not exhibit significant cardiac dysfunction, PKA signaling was impaired and cardiac reserve was attenuated in response to inotropic stimulation. The mice also consistently showed a reduced exercise capacity compared with littermate controls [34]. Given the role of cardiolipin in sustaining mitochondrial respiration and balancing mitochondrial dynamics, its downregulation due to a decrease in the level and quality of lipin is an important mechanism of metabolic inflexibility in muscles [35].

While patients with an *LPIN1* mutation exhibit recurrent rhabdomyolysis from childhood [36, 37], there are no significant myocardial damages. In two children who suffered from an episode of rhabdomyolysis and likely died from cardiac arrest, electrocardiograms showed diffuse T-wave elevations with normal blood potassium. However, their autopsies did not exhibit lethal pathologic changes in the heart [38]. In fact, data on changes in cardiac function of lipin 1-deficient patients varied, with some authors finding a decrease in cardiac output in patients with fever, while others found an increase [39]. In a recent study of eight patients with an *LPIN1* mutation [39], one patient was found to have mild systolic dysfunction during exercise and an exacerbation six months later. Patients presented with intracardiac lipid accumulation that was hypothesized to precipitate toxicity in cardiomyocytes. Although there was variation in these results, all of the evidence supported a diminution in oxidative metabolic capacity in the patients’ skeletal muscle in the presence of environmental stressors [39].

Heart lipin 1 expression can be positively or negatively regulated in response to physiologic stimuli, thereby regulating fatty acid metabolism [27]. In studies on mice, lipin 1 expression was depicted as downregulated in pathologic cardiac hypertrophy and heart failure [27]. A similar situation was observed in human heart failure specimens, suggesting bidirectional interactions between lipins and the cardiovascular system [34].

Collectively, these studies suggest that lipin-1 disturbances alter lipid composition and mitochondrial dysfunction in the heart directly and indirectly, subsequently leading to inflexibility to stress and a tendency toward maladaptation during periods of increased workload. Nonetheless, current studies have mainly focused on the PAP activity of lipins, and the coactivation activity of lipins in the heart has not been elucidated.

Lipin 1 is the major form of lipin in skeletal muscle and heart, and it is hypothesized that lipin 1 may play some similar roles in both of these muscle tissues. Lipin 1 PAP activity regulates autophagy homeostasis in the muscle by catalyzing the production of DAG to promote the maturation of autolysosomes [40] and by mediating Bnip3-LC3 interaction in mitophagy [41]. Loss of lipin1 PAP activity in muscle leads to progressive myopathy with decreased autophagy activity, the accumulation of structurally abnormal mitochondrial and cellular cardiolipin, the increased β-oxidation of fatty acids, and increased reductive demands [42, 43]. A study recently found that treatment with low-concentration hydroxychloroquine sulfate improved physical performance and resting cardiac function in lipin 1-deficient patients, which is postulated to be related to decreased oxidative stress observed in myoblasts [44]. Thus, there may be similar influences on autophagy in cardiomyocytes. Given the scarcity of evidence in humans in this area, further research is necessitated to corroborate these processes and to decipher the underlying mechanisms more clearly.

### Lipins in adipose tissue: adipose dysfunction and systemic metabolism

Unhealthy adipose tissue exerts adverse impacts on systemic metabolism such as insulin resistance, increased inflammation, and altered adipokine secretion–leading to an elevated susceptibility to cardiovascular disease [45].

Lipin 1 is abundantly expressed in adipose tissue and plays a key role in lipid turnover [46], adipocyte differentiation [47], and normal functioning of mature adipocytes [48]. Adipose lipin 1 can promote TAG synthesis via enzymatic activity and regulate lipolysis by (1) regulating the amount of PA and then the activity of phosphodiesterase 4 or (2) promoting the expression of FAO genes through transcriptional coactivation activity [49]. In addition, lipin 1 regulates adipocyte differentiation and adipogenesis, which overlaps with some of the functions of SREBP [18, 20, 50], suggesting that some effects may be in part due to the lipin 1-SREBP interaction; however, the specific process is largely unknown. While *fld* mice manifested lipodystrophy and insulin resistance, specific overexpression of lipin 1 in adipose tissue promoted insulin sensitivity and a drop in blood glucose despite increased adiposity [51], indicating lipin’s role in regulating systemic metabolism via adipose tissue. Indeed, lipin 1 is a negative regulator of inflammatory cytokine secretion, and it exerts its effects by directly repressing NFATc4 [19], while inhibition of lipin 1 expression increases chemotactic protein-1 expression in monocytes, as well as adipose inflammation and systemic insulin resistance [52]. Additionally, in recent studies, investigators have begun to explore the relationship and significance of lipins and adipokines. The levels of leptin and adiponectin were decreased in *fld* mice [9], with a more prominent decrease in the level of adiponectin, which is anti-inflammatory and exhibits a protective effect on the cardiovascular system [53]. Thus, these studies suggest that an adequate amount of lipin in adipocytes may assist in preventing metabolic abnormalities and the inflammatory state.

No clinically significant lipodystrophy was observed in patients with a lipin 1 defect [14], and this was not due to compensation by lipin 2 or lipin 3 [54], indicating the existence of alternative pathways that are important for TAG synthesis in human adipocytes. Several studies have linked lipin expression in adipose tissue to metabolic traits. Lipin 1 expression in adipose tissue displayed a negative association with BMI, insulin resistance [54–56], serum TAG, and leptin levels [57, 58], indicating a possible relationship between adequate level of adipose lipin 1 and advantages in glucose and fatty acid disposal [55, 57]. Other studies, however, found the association between *LPIN1* single-nucleotide polymorphisms (SNPs) and metabolic traits to be inconsistent [59, 60]. A recent study showed that *LPIN1* expression was diminished in individuals with metabolically unhealthy obesity and that it correlated not only with insulin sensitivity but also with hepatic lipogenesis [53]. Moreover, *LPIN1* SNPs are found to be associated with blood pressure. A previous study revealed that male minor allele carriers of rs10495584 had lower mean systolic and diastolic blood pressures than male noncarriers, which is consistent with the findings of another study in which a significant effect was only seen in men [61, 62]. Recent studies have suggested that *LPIN1* may constitute one of the candidate targets for human hypertension and showed that elevated blood pressure and accelerated heart rate in *fld* mice were associated with reduced levels of adipokines such as leptin and adiponectin, along with sympathetic nervous system activation [63].

In summary, these studies imply that abnormalities in lipin expression affect the adipose function and systemic metabolism, predisposing the cardiovascular system to an unhealthy state. It should be noted that different fat depots may exert different effects on the entire system, and additional study is required to discern which components are more critical in terms of cardiovascular health.

### Lipins in the liver: abnormalities in lipid metabolism

Liver metabolism and cardiovascular health are closely linked. Fatty liver diseases involve dysregulated lipid metabolism, elevated systemic inflammation, insulin resistance, endothelial dysfunction, and increased oxidative stress; and interact with adipose tissue to enhance the risk of CVDs [64–66]. Among the relevant elements, dyslipidemia is considered to be a major risk factor in CVDs, and associated components include low-density lipoprotein (LDL) cholesterol, lipoprotein(a), TAG, and high-density lipoprotein (HDL).

In addition to altering PAP enzymatic activity, lipin 1 directly activates the PGC1α/PPARγ pathway to regulate several hepatic metabolic pathways involved in FAO and indirectly inhibit SREBP to repress the expression of lipogenic genes, despite relatively low levels in the liver [4, 18].

The liver phenotype of *fld* mice is largely due to lipid dysregulation. Although neonatal *fld* mice developed transient hypertriglyceridemia and lipin 1 activation led to a significant reduction in circulating TAGs by reducing hepatic TAG secretion and increasing β-oxidation [4], some studies showed a diminution in plasma TAGs in the absence of lipin 1 [33]. Despite having elevated atheroprotective HDL, *fld* mice fed an atherogenic diet exhibited insulin resistance and were more likely to develop atherosclerosis [32]. However, *fld* mice exhibited hepatic PAP activity that was comparable to the wild-type mice, likely due to compensatory upregulation of lipin 3 protein [5]. Similarly, an upregulation of lipin 1 and lipin 3 in the liver was observed in lipin 2-deficient mice as a compensatory mechanism and resulted in elevated hepatic lipid accumulation on a high-fat diet [67]. Mice with lipin 3 haploinsufficiency in their hepatocytes exhibited hypertriglyceridemia, and this haploinsufficiency also induced the overexpression and abnormal distribution of lipin 1 in the cytosol and nucleoplasm [28]. In addition, it was demonstrated that the transcriptional-regulatory function of lipin 1 was responsible for the modulation of hepatic VLDL-TAG secretion [68]. Moreover, lipin 1 deficiency was found to disturb phospholipid metabolism by affecting spliceosome components and upregulating alternative mRNA splicing in the liver during the fasting state, indicating a possible new direction for the in-depth exploration of lipin functions [69]. The above findings suggest that each lipin family member may have unique and irreplaceable roles in regulating liver lipid metabolism. While lipin PAP activity is mainly responsible for TAG synthesis, a lack of coregulation role and downstream gene expression dysregulation are probably the major reasons for excessive lipid accumulation and secretion in lipin-deficient liver.

In addition to alterations in lipid metabolism, studies support the concept that perturbed levels of lipin 1 lead to hepatic inflammation, which is to some extent related to the inhibitory effect of lipin 1 on NFATc4 and NF-κB [70] and hepatic insulin resistance [71–73]; however, it is unclear as to how much they contribute to the systemic CVD-prone state [74]. Thus, lipin 1 may affect cardiovascular health through multiple mechanisms. In this regard, the cross-talk between the liver and other tissues may be important in regulating the metabolic and inflammatory states of the entire body, and we posit that animal models with tissue-specific gene knock-outs facilitate a better understanding of this phenomenon in the future. Notably, although significant dyslipidemia was observed in *fld* mice [32], human patients with *LPIN1* mutations appeared to have a normal lipid spectrum and did not exhibit insulin resistance or liver steatosis [36]. However, due to the relatively young ages of patients, the effects of lipin 1 deficiency on metabolism and subsequently on cardiovascular system functioning in the long term remain unclear. While a diversity of lifestyles and different dietary structures might impact the risks of CVDs via lipin-related mechanisms, more research is needed to elucidate the roles for lipins in liver function under different circumstances and their clinical implications.

### Lipins in macrophages: inflammation and atherosclerosis

Monocyte-derived macrophages are significant in atherosclerosis via the uptake of lipoproteins and by initiating foam cell formation. External environmental and intrinsic factors also regulate macrophage metabolism, causing them to exhibit pro- or anti-inflammatory phenotypes that influence the onset and progression of atherosclerosis [75].

Several studies have implicated lipins 1 and 2 in the inflammatory states of macrophages. Lipin 1, specifically lipin 1α, has been demonstrated to regulate the size and number of lipid droplets, and promote the activation of cytosolic phospholipase A2-α (cPLA2α) and downstream signaling in human macrophages treated with oleic acids [76]. In both mouse and human macrophages, lipin 1 functions in a pro-inflammatory fashion during TLR-signaling activation induced by LPS [77]. Lipin 2 appears to serve a more important role in TAG synthesis of macrophages and possesses an anti-inflammatory capability, as in lipin 2-depleted human blood monocyte-derived macrophages IL-6, MCP-1, and TNF are upregulated upon palmitic acid-stimulation, likely due to impaired TAG synthesis, excess fatty acids, and subsequent activation of the JNK-c-Jun pathway [78]. Other investigators also found that lipin 2 limited LPS-induced IL-1β production by inhibiting the activation of NOD-like receptor family pyrin domain-containing 3 (NLRP3) [79] and mitogen-activated protein kinase (MAPK) [80]. There was evidence supporting the anti-inflammatory properties of lipin 2 in immune cells in a recent study where lipin 2 protected against microglial NLRP3 inflammasome-mediated neuroinflammatory responses in diabetic encephalopathy in mice by inhibiting JNK and ERK pathways [81].

In research on atherosclerosis, lipin 1 was found to be enriched in macrophage-rich regions in human atherosclerotic plaques, and a study in mice confirmed the promoting activity of lipin 1 in LDL-elicited foam cell formation and pro-inflammatory cytokine production [82]. Further research in mice showed that PAP activity of lipin 1 was atherogenic as it enhanced the DAG-dependent PKC-PKCα/βII-ERK1/2-c-Jun-signaling cascade upon stimulation with oxidized LDL or acetylated LDL, thus contributing to a proinflammatory phenotype in macrophages; while inhibiting lipin 1 led to inhibited atherosclerosis [83]. Intriguingly, the transcriptional coregulator function of lipin 1 appeared to exact contradictory actions in macrophage inflammation and to attenuate atherosclerotic plaque progression, allowing an increase in β-oxidation and oxidative phosphorylation and directing macrophages toward a pro-resolving state and continuous efferocytosis during IL-4 stimulation [84]. In mice with myeloid-specific deletion of lipin 1 and on a high-fat diet, the circulating pro-atherogenic cytokines IL-23, IFN-γ, and IL-1β were augmented; while loss of lipin 1 transcriptional coregulatory activity led to an increase in atherosclerotic plaque size and core necrosis size, suggesting its atheroprotective role [85]. The transcriptional coregulator activity of lipin 1 has been shown to play a role in IL-4 mediated macrophage wound-healing [86]. Given that IL-4-mediated polarization of macrophages toward M2 activity promotes plaque stability and as there is a similarity between wound healing and atherosclerotic plaque healing [87], lipins may also be important in the latter healing process.

Lipin 1 maintains a bidirectional position in the regulation of macrophage phenotype, and together with lipin 2 and other members of regulated lipid metabolism, affects DAG- and PA-related signaling pathways or inflammation-associated gene transcription to exert pro- or anti-inflammatory effects, with the net effect depending upon the direction of tilt when equilibrium is achieved. Distant inflammation can also accelerate atherosclerotic plaque progression; it is still plausible that circulating macrophages reach distant sites and alter systemic inflammation that contribute in turn to vascular damage. Currently, there is no evidence that lipin 1 abnormalities in macrophages can lead to a shift in systemic cytokines during wound healing [86]; thus, the influence of lipin 1 on the whole body remains to be determined.

Nevertheless, it is important to note that other types of cells involved in the initiation and progression of atherosclerosis (such as endothelial cells, vascular smooth muscle cells, and other types of immune cells) are equally important. Thus, the functions of lipins in these components and the roles of lipins 2 and 3 require further examination. In addition, there are some questions open for discussion. For example, do the phenotypic alterations in macrophages found in experimental animals also exert a substantial impact on the pathogenesis of atherosclerosis in humans?

## Lipin regulation

The significance of lipins involved in crucial physiologic processes has sparked interest in exploring how lipin expression and activity are regulated. Lipin action is regulated at multiple levels, including the transcription of genes encoding lipins, post-translational modifications, and subcellular protein localization of lipin proteins. This bewildering array of regulatory mechanisms reflects the fact that the regulation of lipins is vital for adjusting to complicated pathophysiologic processes.

### Transcriptional regulation: regulation of genes that encode lipins

Lipin proteins are encoded by different genes, and they vary in their expression patterns and functions within diverse tissues [9]. During the past decades, researchers have explored the role of lipin 1, the principal lipin protein expressed in cardiac tissue, in cardiovascular diseases by elucidating its regulatory mechanism, revealing that lipin 1 is regulated in response to physiologic and pathologic stimuli that affect cardiac metabolism [5, 27]. This review is therefore focused predominantly on the regulation of the gene encoding lipin 1 (*LPIN1*). *LPIN1* responds sensitively to transcriptional stimuli, and many specific factors have been uncovered that regulate gene-expression levels.

Glucocorticoids induce the transcription of *LPIN1* during adipocyte differentiation via the glucocorticoid response element (GRE), a specific transcription factor-binding site in the proximal promoter region of *LPIN1* [88]. Bernard et al. reported that the levels of lipin 1 mRNA were increased by glucocorticoids in neonatal rat cardiomyocytes [16]. In hepatocytes, dexamethasone increases levels of lipin mRNAs [88], thus leading to elevated lipin 1 synthesis and PAP activity. Glucocorticoids might also impact splicing since lipin 1β is induced more strongly than lipin 1α in response to dexamethasone [88]. Insulin counteracts the stimulatory action of dexamethasone on *LPIN1* expression while glucagon or cAMP elevates it [89]. In vivo, conditions such as obesity and fasting promote glucocorticoid levels and also contribute to elevated levels of lipin 1 mRNA in adipose tissue [88].

The expression of *LPIN1* is additionally regulated by cellular sterols through the sterol-response element and nuclear factor Y-binding sites in the human *LPIN1* promoter and is mediated by SREBP-1 and nuclear factor Y [90]. Chae et al. [91] demonstrated that exposure to UVB radiation inhibited lipin 1 expression via SREBP-1 in normal human epidermal keratinocytes (NHEKs), which protects against UVB-induced proinflammatory reactions, and thus assists in resisting photoaging. Ethanol also enhances the expression of *LPIN1* by suppressing AMPK and promoting SREBP-1 [92]. Hypoxia has been found to induce the expression of *LPIN1* via HIF-1α, which binds to a single distal HRE site (a hypoxia-responsive element in the promoter of *LPIN1*) and triggers its activation under low-oxygen conditions [93, 94], and the HIF1α-lipin 1 pathway is inhibited by mitochondrial sirtuin 3 (SIRT3) [95].

The expression of *LPIN1* is modulated by the activity of several transcription factors. The transcription of *LPIN1* in ventricular myocytes was demonstrated to be under the control of estrogen-related receptors (ERRs) and their coactivator PGC-1α [72]. The ERRs in the form of ERRα/γ are associated with an ERR element 1 (ERRE1) in the first intron of *LPIN1* [72]. A recent study showed that injection of clenbuterol, a β2-adrenergic agonist, inhibited the expression of PGC-1α and lipin 1; whereas down-regulation of PGC-1α, ERRα, ERRγ, and lipin1 was observed in failing hearts where the activity of cardiac phosphatidic acid phosphohydrolase was also reduced [27]. HNF4 was also proven to activate the expression of *LPIN1* in concert with PGC-1α through a nuclear receptor response element (NRRE) in the first intron of *LPIN1* in hepatocytes [96]. Moreover, NF-E2-related factor 1 (Nrf1) was identified as a regulator of *LPIN1* through binding to antioxidant response elements (AREs) [97].

Apart from the stimulators mentioned above that promote *LPIN1* transcription, there are several inhibitors of the expression of *LPIN1* that include small heteromeric partner (SHP), lipopolysaccharide (LPS), and pro-inflammatory cytokines such as tumor necrosis factor-α (TNF-α), interleukin-1β (IL-1β), and interferon-γ (IFN-γ) [98, 99]. In cultured 3T3-L1 adipocytes, the expression of *LPIN1* was inhibited by TNF-α, which was neutralized by suppressing Jak2 signaling via the Jak2 inhibitor AG490 [100]. Also, in 3T3-L1 adipocytes, lipin 1 mRNA and protein expression was hindered by the endoplasmic reticulum (ER) stress inducer tunicamycin, and this was reversed by the activation of PPAR-γ [101]. Furthermore, LPS and zymosan impeded *LPIN1* expression in mouse adipose tissue through the activation of the Toll-like receptors TLR4 and TLR2, respectively [99]. A recent study on how the mechanical stimulation of extracellular matrix (ECM) regulated lipid metabolism revealed that attenuated actomyosin contractility caused the inhibition of lipin 1 [102].

MicroRNAs, a class of small non-coding RNAs that regulate gene expression by binding to the 3′-untranslated region (UTR) of target mRNAs, also indirectly regulate *LPIN1* expression. The function of hepatic sirtuin 1 (SIRT1) was reduced by ethanol, a significant factor in the development of alcoholic fatty liver disease. Several investigators identified miR-217 as improving ethanol-mediated fat agglomeration both in cultured mouse AML-12 hepatocytes and in the livers of mice who were fed ethanol continuously [103]. By down-regulating SIRT1, miR-217 regulates ethanol-induced hepatic inflammation by interfering with the miR-217-sirtuin 1-lipin 1 axis, performing an essential role in macrophage-dependent inflammation in response to ethanol- and LPS-induced injury [104, 105]. Additionally, miRNA-1914-5p modulates the expression of lipin 1 in LX-2 cells, a universally adapted immortal human stellate cell line [106]. In 2019, Zhao et al. [107] observed that miR-451a exerted its anti-tumor effect by inactivating lipin 1 in the endothelial and neoplastic cells of hepatocellular carcinoma (HCC).

Another notable issue is the interaction among different members of the lipin family at the transcriptional level. Although PAP activity exists for lipin 2, it is lower than that of lipin 1 [26]. However, in contrast to lipin 1, the expression of lipin 2 was not modulated by stimuli such as glucocorticoids, PGC-1α, or cAMP signaling [108]. In HeLa cells, e.g., silencing of lipin 2 resulted in an increase in mRNA and protein levels as well as in the PAP activity of lipin 1. In differentiating 3T3-L1 adipocytes, siRNA knockdown of lipin 1 enhanced lipin 2 expression levels commensurately [108]. These studies have provided new insights into the latent relationship between lipin 1 and lipin 2.

### Post-translational regulation: protein modification and subcellular localization

**Fig. 3:**
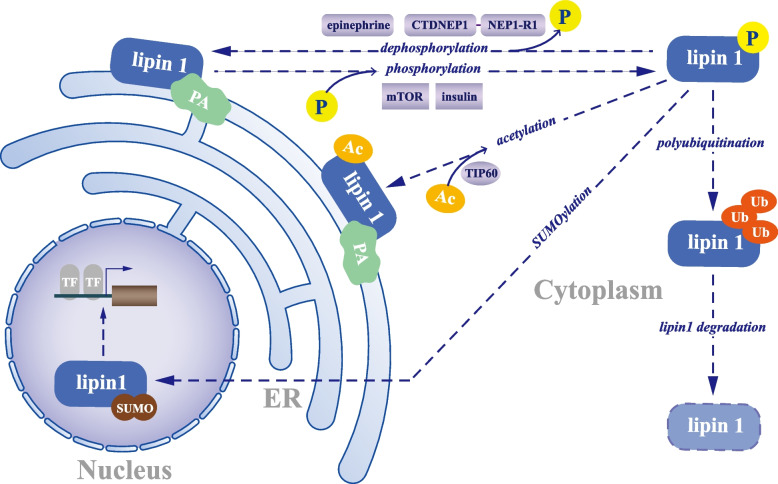
Post-translational regulation of lipin1. Post-translational modifications of lipin 1 include phosphorylation, SUMOylation, acetylation, and ubiquitination. Phosphorylation promoted by insulin and mTOR triggers the translocation of lipin 1 protein from the ER membranes to the cytoplasm. The CTDNEP/NEP1-R1 phosphatase complex and epinephrine then dephosphorylate lipin 1 and promote its membrane association, allowing lipin 1 to react with its substrate to produce DAG. The membrane association of lipin 1 is also induced by acetylation via TIP60 acetyltransferase. The modification of lipin 1 by SUMOylation preferentially promotes lipin 1 nuclear localization, and polyubiquitination facilitates the degradation of lipin 1

Post-translational modifications of lipin proteins include phosphorylation, SUMOylation, acetylation, and ubiquitination [18, 30, 109–115] (Fig. 3). A leading mechanism underlying the regulation of lipin 1 PAP activity is through reversible protein phosphorylation. PAP activity remains in the cytosol until it is mobilized to the ER membrane in response to a sequence of cues. Harris et. al determined that phosphorylation was implicated in PAP activity by regulating the subcellular localization of lipin 1 because the depressor of oleic acid or epinephrine promoted lipin 1 dephosphorylation and translocated it to the ER membrane [30].

It is important to indicate that phosphorylation does not directly alter the intrinsic PAP activity of lipin 1, but rather reduces the physiologic expression of PAP activity by altering its subcellular location [30]. Direct evidence behind phosphorylation and dephosphorylation in regulating the displacement of lipin 1 is examined below. In contrast to lipin 1 phosphorylation (which determines its membrane association, thus affecting its PAP activity), the other two lipin family members do not negatively regulate either membrane binding or PAP activity when phosphorylated [109, 116].

As noted earlier, the dephosphorylation of lipin 1 leads to its translocation from the cytosol to the karyotheca and ER membranes to produce DAG [116, 117]. The CTDNEP1/NEP1-R1 phosphatase complex then dephosphorylates lipin 1 and facilitates its membrane association with the ER to permit lipin 1 to contact its substrate, PA [111, 117]. Subsequently, Harris et al. demonstrated that epinephrine and oleic acid promoted dephosphorylation of lipin 1 without affecting PAP activity [30].

In contrast, the phosphorylation of lipin 1 might also be activated through insulin, thus resulting in the cytosolic localization of lipin 1; and this depends upon phosphatidylinositol-3-kinase activity and the mammalian target of rapamycin (mTOR)-signaling pathways [18, 30, 110, 118]. Highly phosphorylated lipid 1 binds to 14–3-3 proteins and resides in the cytoplasm [114]. Using cell-based and in vitro experiments, Hiroko et al. [119] identified a novel regulator of lipin 1 referred to as PGAM5 that could directly dephosphorylate lipin 1. Furthermore, carbonyl cyanide m-chlorophenyl hydrazone (CCCP), a compound that triggers endogenous PGAM5, induces lipin 1 dephosphorylation and translocation to the nucleus [4].

Furthermore, many studies have investigated the possible functions of dephosphorylated lipin 1 located in the nucleus, such as its role as a regulator of transcription factors [4] and its effects on SREBP, providing an insight into the role of lipin 1 as a key component of the mTORC1-SREBP pathway [18].

To summarize, dephosphorylation by oleic acid, epinephrine, and the CTDNEP1/NEP1-R1 phosphatase complex induces translocation of lipin 1 protein and PAP activity from the cytoplasm and attaches it to organellar membranes, whereas phosphorylation by insulin produces the opposite effect. Beyond phosphorylation, lipin 1 is also modified by SUMOylation, which promotes nuclear localization of lipin 1 in cultured neuronal cells [113]. Furthermore, the acetylation of TIP60 acetyltransferase stimulates the binding of lipin 1 to membranes [112]. Conversely, polyubiquitination advances the degradation of lipin 1 [110].

In addition, it is worth noting that the roles of lipins 2 and 3 cannot be ignored even though few studies have probed their regulation, since they are capable of ameliorating the effects of lipin 1 by compensating for its PAP activity [5, 120]. Thus, it is necessary to further scrutinize lipin regulation to deepen our understanding of the roles of lipins in the progression of CVD.

## Clinical implications

As noted above, the impacts of lipins in modulating heart homeostasis and activity have been broadly explored in patients with *LPIN1* mutations [38, 39] and mouse models [121, 122]. This implies that some drugs target CVDs act by regulating the expression and activity of lipins. Herein, we provide a relatively comprehensive introduction to lipins in the context of CVDs and shed light on the clinical significance of lipin biology.

Some investigators have determined that several medications prescribed in CVDs—such as statins, dihydroartemisinin (DHA), and propranolol—appear to be associated with lipins. Statins—frequently prescribed and well tolerated as drugs used to lower cholesterol—are regarded as the most powerful pharmaceuticals applied for the treatment of hypercholesterolemia, the decisive risk factor in atherosclerosis [123, 124]. The serious side effects of statins lie in myotoxicity, which is manifested as myopathy, myalgia, myositis, and rhabdomyolysis. A previous study has reported that approximately 1–5% of statin users experience muscle symptoms, a small group of whom even suffer rhabdomyolysis, which may result from a variety of pathophysiologic mechanisms [125]. Mounting evidence has demonstrated that genetic variations may be responsible for statin-related adverse effects in certain individuals [126]. Of these, heterozygous *LPIN1* missense mutations can facilitate myopathy induced by statins [37, 127].

Zhang et al. [40] reported that lipin 1 mediated autophagy clearance and interacted with the pharmacodynamics of statins in skeletal muscle, and that lipin 1-mediated repair contributed to curtailing the myotoxicity of statins in vivo. In addition, Zhang et al. provided possible explanations for major side effects caused by statins, and they found that statin application affects muscle lipid concentrations and exacerbates the effects of lipin 1 haploinsufficiency, further impairing mitochondrial function and autophagy in muscle. Additionally, they revealed the mechanism behind myopathy induced by attenuated lipin 1 activity in response to statin therapy and identified the specific intersection of lipin 1 deficiency and statin pharmacodynamics.

Previous research depicted DHA as able to alleviate alcoholic fatty liver by regulating lipin 1 signaling via the inhibition of the ER stress-JNK/CHOP-mitochondrial cascade. Bao et al. [128] very recently confirmed that DHA, a type of artemisinin derivative, relieved pulmonary hypertension and vasoconstriction in rodent models, shedding light on future clinical drug research and development aimed at treating CVDs related to lipin regulation.

The elevated concentrations of plasma TAG are confirmed to constitute a potent marker of cardiovascular disease, and strategies to lower plasma TAG levels have been proven to alleviate cardiac dysfunction [121]. PPARα agonists such as clofibrate and gemfibrozil are therapeutic agents that have been shown to diminish serum TAG-rich lipoprotein levels, thus being critical to the treatment of atherosclerosis [129]. Intriguingly, lipin 1 has been demonstrated to be an activator of PPARα [130], suggesting that it constitutes a potential therapeutic target for atherosclerosis. It is therefore of paramount importance to further investigate the clinical applications of lipins in CVDs.

We described previously that normal physiologic function of the heart depends upon appropriate lipid metabolism [29], and its important elements include insulin resistance, elevated inflammation, and altered adipokine secretion that may increase the risk of cardiovascular disease [45]. We therefore expect a growing number of enzymes involved in lipid synthesis to become potential targets in the development of anti-CVDs therapies.

## Conclusions

**Fig. 4:**
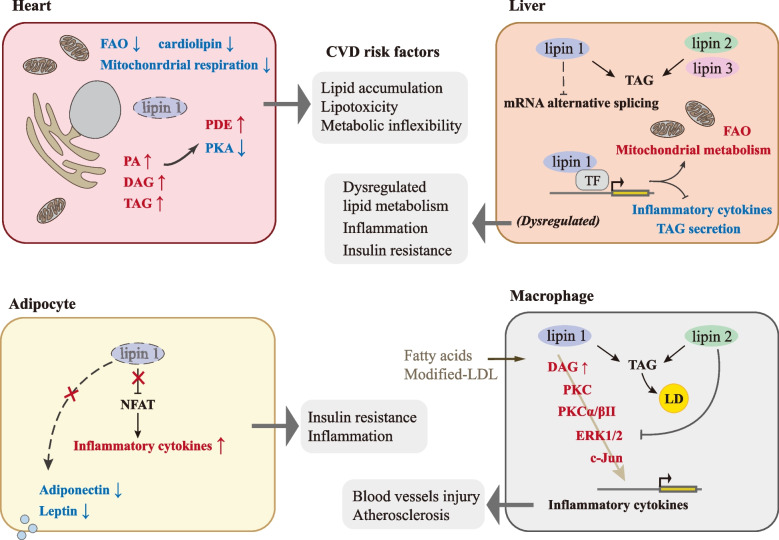
The role of lipins in the pathophysiology of CVDs. A normal quantity and quality of lipins are important for maintaining a balance in lipid metabolism and for the orderly functioning of related signaling pathways in the heart, adipose tissue, liver, and macrophages; perturbation of these processes then contributes to the pathology underlying CVDs. A diminution in lipin 1 in cardiomyocytes is specifically associated with reduced cardiac function and heart maladaptation during stress. Lipin 1 portrays a major role in adipose tissue, and defects in the molecule are related to dyslipidemia, systemic insulin resistance, elevated inflammation, and adipokine disturbances. Intriguingly, in macrophages, lipin 2 appears to be anti-inflammatory while lipin 1 appears to be pro-inflammatory. In the liver, lack of lipin 1 or 2 is related to dysregulation of hepatic lipid metabolism and inflammation, while excessive lipin 1 or 2 induced by some triggers leads to hepatic insulin resistance. However, ascertaining whether alterations in the liver exert a direct influence on the cardiovascular system, and identifying the mechanism(s) by which lipins govern the regulation of other cardiovascular components, remain unresolved

In this study, lipins were found to be important regulators of lipid synthesis and fatty acid oxidation balance in the liver, adipose tissue, skeletal muscle, heart, macrophages, and many other tissues, in addition to affecting cellular lipid-metabolic and related signaling pathways. In terms of the cardiovascular system, lipins acted directly by participating in the pathophysiologic processes of cardiovascular disease, or by interacting with the liver, adipose tissue, macrophages, and other related tissues and cells. In many scenarios, deviations from normal lipin levels appeared to exert little effect on human cardiovascular health under normal conditions but rather led to inflexibility and maladaptation during stress (Fig. 4). While lipin dysregulation was related to CVDs, lipins appeared to behave reciprocally with some physiologic or pathologic conditions in the cardiovascular system. Recent studies have revealed that some drugs affect the expression and action of lipins, and these may provide a reference for mining underlying mechanisms of action and provoking the development of novel anti-CVD drugs. However, little is known regarding the role(s) lipins occupy in the cardiovascular system itself, so further research in this area is needed to elucidate their position in cardiovascular health and disease.

## Data Availability

Not applicable.

## Abbreviations

AREs: Antioxidant response elements
CCCP: Carbonyl cyanide m-chlorophenyl hydrazine
CDP-DAG: Cytidine diphosphate diacylglycerol
CDS: CDP-DAG synthase
C-LIP: Carboxy-terminal lipin
cPLA2α: Cytosolic phospholipase A2-α
CVDs: Cardiovascular diseases
DAG: Diacylglycerol
DGAT: Diacylglycerol acyltransferase
DGK: Diacylglycerol-kinases
DHA: Dihydroartemisinin
ECM: Extracellular matrix
ERRE1: ERR element 1
ERRs: Estrogen-related receptors
FAO: Fatty acid oxidation
*Fld*: Fatty liver dystrophy
GPAT: Glycerol phosphate acyltransferase
GR: Glucocorticoid receptor
GRE: Glucocorticoid response element
HCC: Hepatocellular carcinoma
HDL: High-density lipoprotein
HNFα: Hepatic nuclear factor α
IL-1β: Interleukin-1β
IFN-γ: Interferon-γ
LDL: Low-density lipoprotein
3’-UTR: 3’-Untranslated region
LPAAT: Lyso-PA-acyltransferase
LPS: Lipopolysaccharide
M-LIP: Middle lipin
MAPK: Mitogen-activated protein kinase
mTOR: Mammalian target of rapamycin
mTORC1: MTOR Complex 1
NHEKs: Normal human epidermal keratinocytes
N-LIP: Amino-terminal lipin
NLRP3: NOD-like receptor family pyrin domain-containing 3
NLS: Nuclear localization signal
Nrf1: NF-E2-related factor 1
NRRE: Nuclear receptor response element
PA: Phosphatidic acid
PAP: Phosphatidate phosphatase
PBD: Poly basic domain
PGC-1α: PPAR γ coactivator-1α
PLA: Phospholipase A
PPAR: Peroxisome proliferator-activated receptor
PS: Phospholipid
SHP: Small heteromeric partner
SIRT1: Sirtuin 1
SIRT3: Sirtuin 3
SREBP-1: Sterol regulatory element-binding protein 1
TAG: Triacylglycerol
TNF-α: Tumor necrosis factor-α
TFs: Transcriptional factors
